# Effect of Minocycline Pleurodesis in Infants With Refractory Chylothorax After Palliative Surgery for Complex Congenital Heart Disease

**DOI:** 10.7759/cureus.23506

**Published:** 2022-03-26

**Authors:** Kanchi Saito, Hirofumi Saiki, Shigekuni Tsuchiya, Yurie Takizawa, Akira Sato, Takuya Goto, Yukiko Toya, Atsushi Matsumoto, Junichi Koizumi, Kotaro Oyama, Manami Akasaka

**Affiliations:** 1 Pediatric Cardiology, School of Medicine, Iwate Medical University, Shiwa, JPN; 2 Neonatology, School of Medicine, Iwate Medical University, Shiwa, JPN; 3 Cardiovascular Surgery, School of Medicine, Iwate Medical University, Shiwa, JPN; 4 Pediatrics, Michinoku Center for Disabled Children, Shiwa, JPN; 5 Pediatric Neurology, School of Medicine, Iwate Medical University, Shiwa, JPN

**Keywords:** chemical pleurodesis, palliative surgery, extremely low birth weight infant, pediatric congenital heart disease, chylothorax

## Abstract

Chylothorax is a critical complication after surgery for congenital heart disease, which markedly compromises the postoperative course with increased mortality. As the cardiovascular load additively causes stagnation of the thoracic duct, chylothorax after palliative cardiac surgery can be highly refractory to the therapies. Here we report a case of two patients with refractory chylothorax attributed to hemodynamic load which was successfully treated with minocycline pleurodesis. In combination with congenital heart disease, extremely low birth weight coupled with prematurity in case 1 and venous obstruction with excessive volume load due to additional aortopulmonary shunt in case 2 additively increased resistance to the therapies, including fasting with total parenteral nutrition (TPN), XIII factor supplementation, octreotide infusion, as well as the use of steroids. As pleural effusion was sustained at more than 50 ml/kg/day, the condition of both patients deteriorated severely; pleurodesis using minocycline was urgently introduced. Pleural effusion declined at every session and both cases were in remission in a few sessions without unfavorable acute reaction. No symptoms suspecting chronic adverse effects were observed during follow-up, including respiratory dysfunction, pulmonary hypertension, tooth staining, or abnormal bone mineralization. Although the application of minocycline for children should be minimized, minocycline pleurodesis can be an option for patients with refractory and life-threatening chylothorax.

## Introduction

Postoperative chylothorax markedly compromises hemodynamics, nutritional status, and immunologic condition, by which mortality is heightened and length of hospital stay and medical expenses increase in patients with congenital heart diseases. Particularly in infants after palliative surgery, the venous congestion is easily provoked by the fluctuation of hemodynamic or respiratory condition, where increased thoracic duct pressure have chylothorax that is further resistant to therapies. When upper body venous obstruction is complicated, the mortality increases to more than 20% in neonates and infants, highlighting the strong need for reliable therapeutic intervention [1]. Since access to invasive treatments including thoracic duct ligation or percutaneous lymphatic embolization is limited in neonates and infants, total parenteral nutrition (TPN) or medium-chain triglyceride (MCT) formula, coupled with chemical pleurodesis has been applied to patients with severe refractory chylothorax. While minocycline pleurodesis has been recognized as an effective intervention for chylothorax even in children, its efficacy for patients with unstable hemodynamics, particularly developed after palliative surgery for congenital heart disease, is limited [2-4]. Despite the increasing availability of other chemical agents including talc, accessibility of these agents is often limited depending on the institute, age, body size, and coexisting physical condition, particularly for children. Here we report a case of two infants with refractory chylothorax who were urgently and successfully treated with minocycline pleurodesis.

## Case presentation

Case 1

**Figure 1:**
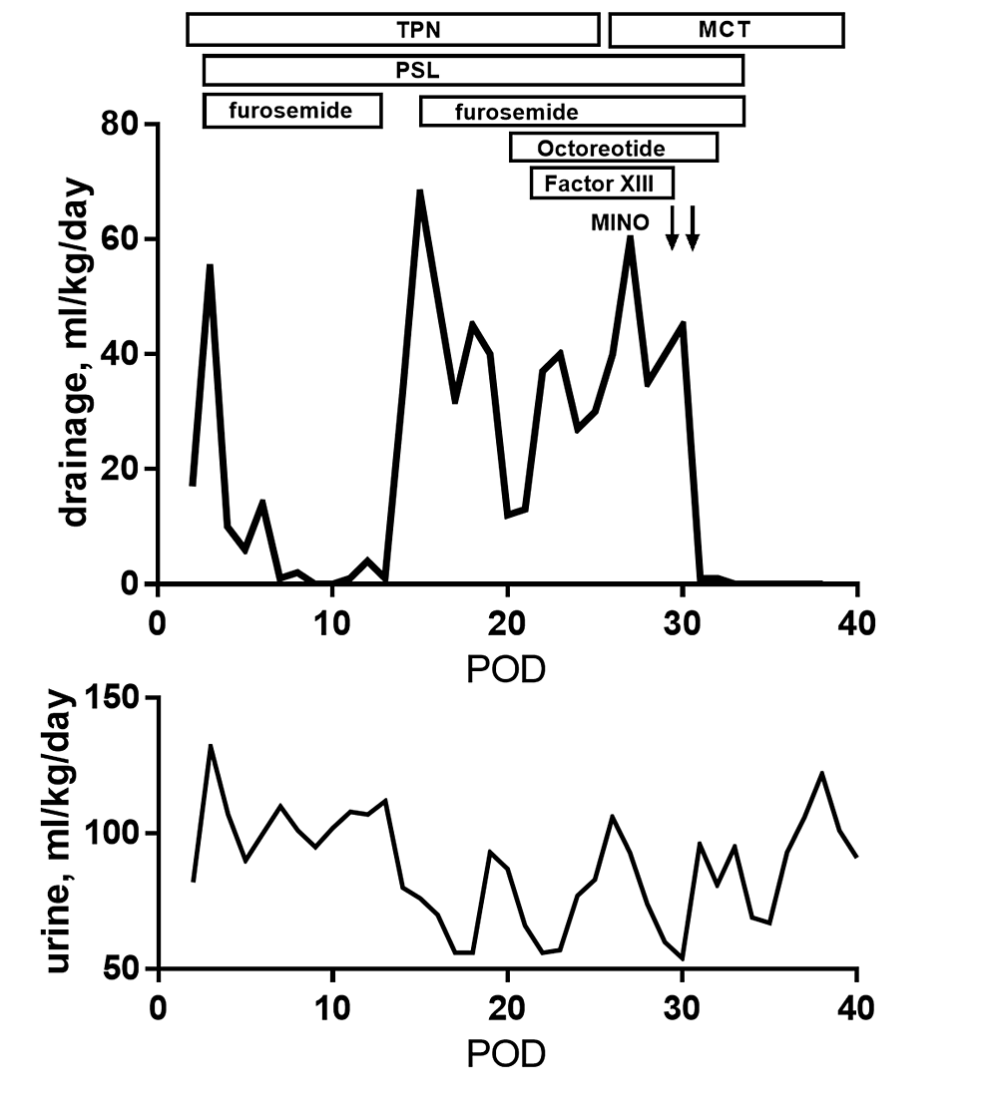
Clinical Course of Case 1 Despite active use of medications, pleural effusion by chylothorax continued, and thus minocycline pleurodesis was performed. MCT: medium-chain triglyceride, POD: postoperative day, PSL: prednisolone, TPN: total parenteral nutrition.

The patient was a baby boy who was delivered by caesarean section due to maternal hemolysis, elevated liver enzymes, and low platelets (HELLP) syndrome at 29 weeks of gestational age, with a body weight of 993 g. At the admission to the NICU, he was diagnosed with respiratory distress syndrome, and intratracheal pulmonary surfactant was administered. An echocardiogram diagnosed him with a double outlet right ventricle (DORV), mild pulmonary stenosis (PS), and patent ductus arteriosus (PDA). As the symptomatic PDA compromised systemic circulation within a few days after birth, PDA ligation was palliatively performed nine days after birth. His respiratory condition acutely declined at postoperative day (POD) 2 and significant pleural effusion in the left chest was detected, which biochemistry indicated as chylothorax, although lung congestion was also suggested by the chest X-ray. Despite multiple therapeutic interventions including continuous chest drainage, TPN, octreotide (5.5 mcg/kg/hr), prednisolone (2 mg/kg/day) as well as XIII coagulation factor supplementation (100 unit/kg/day), pleural effusion continued to increase up to more than 60 ml/kg/day and his condition deteriorated due to malnutrition (Figure 1). Although his pulmonary blood flow had partially suppressed after PDA ligation, mild PS coupled with chronic lung disease markedly varies pulmonary blood flow with the fluctuations of pulmonary resistance. As he was assumed to be severely immune-compromised due to extremely low birth weight with prematurity, and as the massive effusion of more than 60 ml/kg/day was life-threatening, minocycline pleurodesis was urgently introduced with a dose of 8 mg/kg/dose at a maximum of 90 minutes clamping on POD 29 and POD 32, with 72 hours’ interval. His pleural effusion diminished on POD 33 and he was transferred to the local hospital for growing up care without any complications on POD 77. At the age of 10 months, cardiac catheterization revealed mean pulmonary pressure of 20 mmHg and pulmonary resistance of 3.5 WU-m^2^, which was supposed to be optimal as an infant with prematurity induced chronic lung disease. Based on these data, DORV repair with baffle rerouting was performed and no chronic untoward effect was identified including respiratory dysfunction, tooth staining, or abnormal bone mineralization.

Case 2

The patient was prenatally diagnosed with congenitally corrected transposition of great arteries with pulmonary atresia; a modified Blalock-Taussig shunt was constructed at six days after birth. The innominate vein was obstructed during her ICU stay. At the age of five months, a bidirectional Glenn procedure was performed, leaving shunt patent as an additional flow in order to secure pulmonary vascular growth with the occluded innominate vein. She left the ICU uneventfully on POD 6, however, a chest X-ray on POD 10 revealed massive bilateral pleural effusion. Despite active use of diuretics and dietary modification using MCT formula, pleural effusion continued to increase and biochemistry confirmed her with chylothorax.

**Figure 2:**
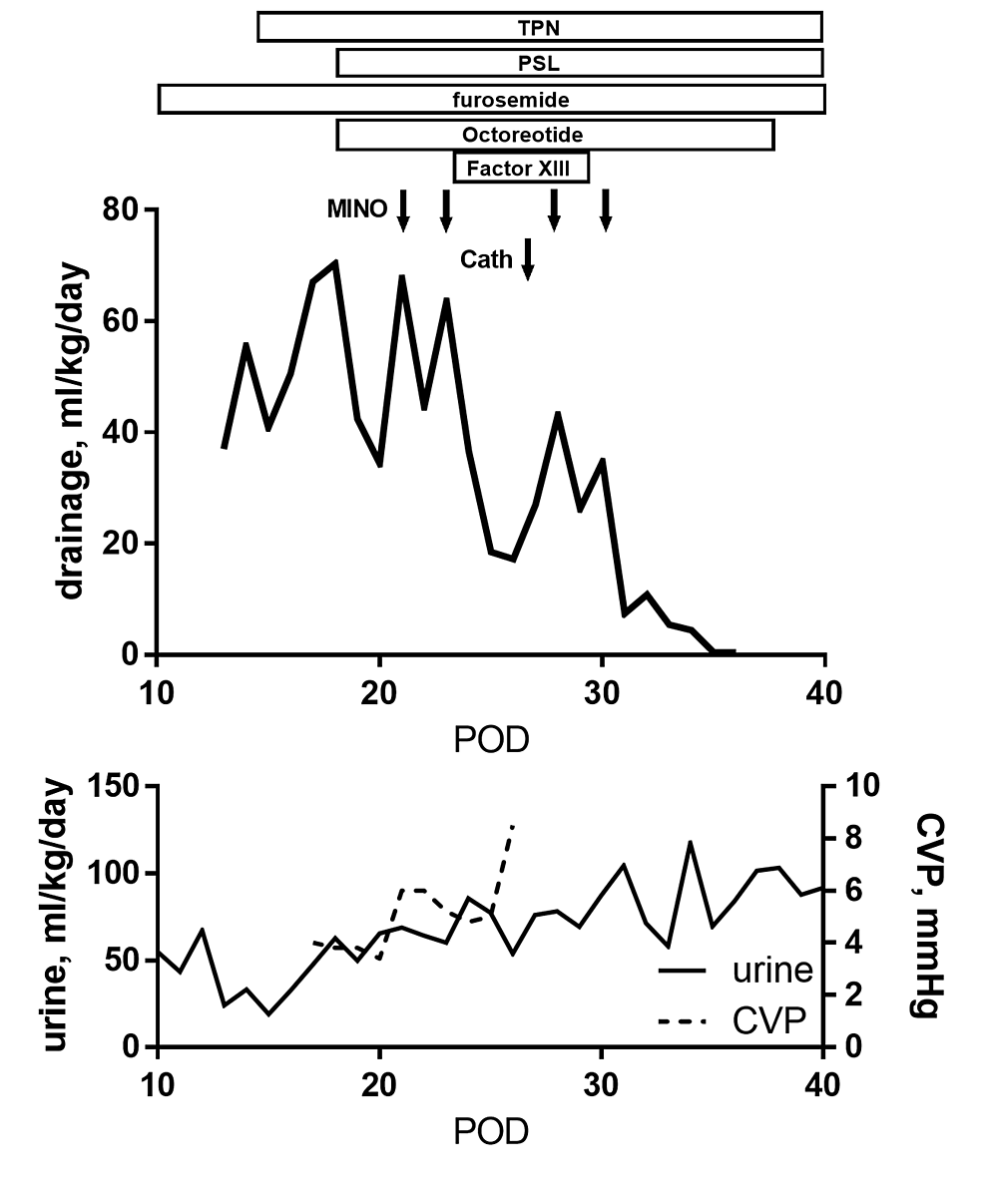
Clinical Course of Case 2 Chylothorax was refractory against multiple less invasive procedures. Although the pleural effusion increased after percutaneous coil embolization for APCAs with the increase of CVP, minocycline pleurodesis successfully brought chylothorax to remission. APCA: aortopulmonary collateral arteries, CVP: central venous pressure measured in the inferior vena cava, MCT: medium-chain triglyceride, POD: postoperative day, PSL: prednisolone, TPN: total parenteral nutrition.

In addition to TPN, intravenous methylprednisolone (2 mg/kg/day), XIII coagulation factor supplementation (30 u/kg/day), and octreotide infusion (4 mcg/kg/hr) were started but it was ineffective. Since propranolol is known to suppress lymphatic flow, we also added it (Hemangiol® syrup, 1.5 mg/kg/day). Despite these medications, pleural effusion of more than 50 ml/kg/day lasted for a week and her urination, as well as serum protein level, declined. Since massive effusion compromised hemodynamics and serum protein level was hardly maintained by active fresh frozen plasma infusion, minocycline pleurodesis for bilateral chest at 4 mg/kg/day was semi-urgently introduced on POD 21 and POD 23, which halved effusion to 20-30 ml/kg/day in both (Figure 2). As the velocity of the aortopulmonary shunt was approximately 4m/sec with a systemic systolic blood pressure of 60-70 mmHg, we assumed superior vena cava (SVC) pressure should be optimal as a Glenn circulation. However, we started to suspect the shunt flow might have been detrimental since blood pressure elevation during wailing increased pleural effusion. Accordingly, we assessed hemodynamic status by cardiac catheterization. While SVC pressure, as well as mean pulmonary pressure, was 11-12 mmHg at first, it markedly augmented to 16 mmHg after contrast imaging with increasing blood pressure from 76/32/49 mmHg to 94/36/62 mmHg, which convinced us that pressures of SVC and thoracic duct would have been strongly affected by systemic pressure and blood volume. As temporal occlusion of Blalock-Taussig shunt suppressed arterial oxygen saturation from 82% to 72%, and as the renal function cannot permit further use of diuretics or systemic vasodilators, we decided to embolize the aortopulmonary collateral arteries (APCA) thoroughly instead of embolizing the shunt. Since diastolic blood pressure was reasonably elevated after embolization, pulmonary vasodilators to maximally expand the pulmonary vascular bed were initiated to suppress fluctuation of venous congestion. Then, the third and fourth dose of minocycline was administered bilaterally, and her chylothorax came to remission after the third dose in the right and after the fourth dose in the left. Her pulmonary vascular growth was favorable in the echocardiogram, and any possible chronic adverse effects were observed including pulmonary hypertension, respiratory distress, tooth staining, or abnormal bone mineralization.

## Discussion

The incidence of postoperative chylothorax in children with congenital heart disease is approximately 3%-9%, with doubled in-hospital mortality [1,5-7]. Among available therapies, percutaneous embolization or surgical ligation of the thoracic duct is increasingly applied even for children [8]. Although promising results are reported, significant barriers exist for applying them to neonates and infants due to small body size and fragility. In addition, patients after palliative surgery still possess burdens of excessive cardiovascular load, thus even a small fluctuation of hemodynamics can provoke worsening of venous congestion. The multi-institution database for children after cardiovascular surgery revealed that 196 (8.9%) among 2205 children who developed postoperative chylothorax required intervention with either pleurodesis or thoracic duct ligation, suggesting limited efficacy of conservative therapies in this cohort [1].

OK-432, which is the inactivated low-virulence strain of Streptococcus pyogenes A3 and induces a strong local inflammatory response, has been used as an adhesive agent for neonates. While successful remission of chylothorax using OK-432 is reported [9], accompanying fever, pain, and elevation of serum inflammatory markers often subject patients to the sepsis workup [10], which has been a significant limitation for compromised patients. Meanwhile, the mechanism of minocycline pleurodesis causes strong acidity which induces acidic damage to the mesothelial cells in the pleural layer, provoking adhesion of the pleural cavity [11,12]. Accordingly, minocycline pleurodesis is relatively independent of the inflammatory response as compared with other agents including OK-432, which would be a significant advantage when treating severely compromised patients. Indeed, Kaneko et al. and Iinuma et al. used 3-26 mg/kg/dose of minocycline and successfully treated congenital chylothorax without any acute adverse events in neonates and infants with structurally normal hearts [2,13]. In compliance with their experience, our patients exhibited neither fever nor elevation of serum inflammatory markers.

While the effect of pleurodesis is considered to be dose-dependent, Masahata et al. reported the successful management of neonatal idiopathic chylothorax as low as 2 mg/kg/dose of minocycline pleurodesis [2,4,13]. As the pH of minocycline solution with concentrations of 6 mg/ml, 2 mg/ml, 1 mg/ml has been reported to be 2.56, 2.84, 3.02, respectively [13], we estimated that a single dose (4-8 mg/kg/dose) of minocycline pleurodesis with 4-6 hours clamping would cause reasonable acidity in our patients even though the pleural effusion constantly increases at the rate of more than 50 ml/kg/day. To suppress the dose of minocycline, supportive therapy is of importance. Since effusion in case 2 continued after the first dose of minocycline, we performed APCA embolization coupled with active use of pulmonary vasodilators (sildenafil 2 mg/kg/day, macitentan 0.2 mg/kg/day) to suppress SVC pressure [1,14]. Although the effusion was not decreased after embolization, improved response with the same dose of minocycline highlighted the importance of suppressing venous pressure to achieve remission [15].

Cascio et al. and Grieco et al. reported in their research that by using minocycline intravenous administration, no significant adverse effect was observed in children younger than eight years old [16,17]. Since massive effusion made patients cachexic, we urgently used minocycline instead of using other agents in these cases. However, regimens for pleurodesis were carefully determined to minimize the possibility of adverse effects. We secured at least a 72-hour interval when repeating administration [18]. Tanaka et al. measured blood concentration of minocycline after a single injection to the hepatic cyst and found that the peak blood concentration was halved compared with that injected intravenously, and it remained for approximately 36 hours [19]. In our patients, pleural effusion mixed with minocycline was removed in no more than 90 minutes, thus, the blood concentration was expected to be further reduced. So far, no chronic adverse event including tooth staining or abnormal bone mineralization was observed. As these infants presented after palliative surgery, the possible adverse effects of pleurodesis on the subsequent procedures should be also considered. While we have not experienced either respiratory dysfunction or augmented pulmonary vascular resistance at this point, a continuous follow-up would be needed. Similarly, the history of pleurodesis may affect future thoracotomy. Taken together, intensive discussion among the cardiovascular team should be implemented before the application of minocycline pleurodesis.

## Conclusions

Minocycline pleurodesis successfully mitigated postoperative refractory chylothorax in patients after palliative surgery for congenital heart disease. Even in highly refractory cases, minocycline pleurodesis aids patients in achieving remission of chylothorax safely. Although full efforts to avoid possible acute as well as chronic side effects should be implemented, minocycline pleurodesis can be an alternative option for high-risk patients with refractory chylothorax.
